# Sodium-Glucose Transport Protein 2 Inhibitor Use for Type 2 Diabetes and the Incidence of Acute Kidney Injury in Taiwan

**DOI:** 10.1001/jamanetworkopen.2023.0453

**Published:** 2023-02-22

**Authors:** Mu-Chi Chung, Peir-Haur Hung, Po-Jen Hsiao, Laing-You Wu, Chao-Hsiang Chang, Kai-Yu Hsiao, Ming-Ju Wu, Jeng-Jer Shieh, Yu-Chuen Huang, Chi-Jung Chung

**Affiliations:** 1 Division of Nephrology, Department of Medicine, Taichung Veterans General Hospital, Taichung, Taiwan; 2 PhD Program in Translational Medicine, National Chung Hsing University, Taichung, Taiwan; 3 Rong Hsing Research Center for Translational Medicine, National Chung Hsing University, Taichung, Taiwan; 4 Department of Medical Laboratory Science and Biotechnology, Asia University, Taichung, Taiwan; 5 Department of Internal Medicine, Ditmanson Medical Foundation Chiayi Christian Hospital, Chiayi, Taiwan; 6 Department of Applied Life Science and Health, Chia-Nan University of Pharmacy and Science, Tainan, Taiwan; 7 Department of Urology, China Medical University and Hospital, Taichung, Taiwan; 8 Department of Public Health, College of Public Health, China Medical University, Taichung, Taiwan; 9 Division of Thoracic Surgery, Tri-Service General Hospital, National Defense Medical Center, Taipei, Taiwan; 10 Institute of Biomedical Sciences, National Chung Hsing University, Taichung, Taiwan; 11 Department of Education and Research, Taichung Veterans General Hospital, Taichung, Taiwan; 12 School of Chinese Medicine, China Medical University, Taichung, Taiwan; 13 Department of Medical Research, China Medical University Hospital, Taichung, Taiwan

## Abstract

**Importance:**

The association between sodium-glucose transport protein 2 inhibitor (SGLT2i) use and the incidence of acute kidney injury (AKI) remains controversial. The benefits of SGLT2i use in patients to reduce AKI requiring dialysis (AKI-D) and concomitant diseases with AKI as well as improve AKI prognosis have not yet been established.

**Objective:**

To investigate the association between SGLT2i use and AKI incidence in patients with type 2 diabetes (T2D).

**Design, Setting, and Participants:**

This nationwide retrospective cohort study used the National Health Insurance Research Database in Taiwan. The study analyzed a propensity score–matched population of 104 462 patients with T2D treated with SGLT2is or dipeptidyl peptidase 4 inhibitors (DPP4is) between May 2016 and December 2018. All participants were followed up from the index date until the occurrence of outcomes of interest, death, or the end of the study, whichever was earliest. Analysis was conducted between October 15, 2021, and January 30, 2022.

**Main Outcomes and Measures:**

The primary outcome was the incidence of AKI and AKI-D during the study period. AKI was diagnosed using *International Classification of Diseases *diagnostic codes, and AKI-D was determined using the diagnostic codes and dialysis treatment during the same hospitalization. Conditional Cox proportional hazard models assessed the associations between SGLT2i use and the risks of AKI and AKI-D. The concomitant diseases with AKI and its 90-day prognosis, ie, the occurrence of advanced chronic kidney disease (CKD stage 4 and 5), end-stage kidney disease, or death, were considered when exploring the outcomes of SGLT2i use.

**Results:**

In a total of 104 462 patients, 46 065 (44.1%) were female patients, and the mean (SD) age was 58 (12) years. After a follow-up of approximately 2.50 years, 856 participants (0.8%) had AKI and 102 (<0.1%) had AKI-D. SGLT2i users had a 0.66-fold risk for AKI (95% CI, 0.57-0.75; *P* < .001) and 0.56-fold risk of AKI-D (95% CI, 0.37-0.84; *P* = .005) compared with DPP4i users. The numbers of patients with AKI with heart disease, sepsis, respiratory failure, and shock were 80 (22.73%), 83 (23.58%), 23 (6.53%), and 10 (2.84%), respectively. SGLT2i use was associated with lower risk of AKI with respiratory failure (hazard ratio [HR], 0.42; 95% CI, 0.26-0.69; *P* < .001) and shock (HR, 0.48; 95% CI, 0.23-0.99; *P* = .048) but not AKI with heart disease (HR, 0.79; 95% CI, 0.58-1.07; *P* = .13) and sepsis (HR, 0.77; 95% CI, 0.58-1.03; *P* = .08). The 90-day AKI prognosis for the risk of advanced CKD indicated a 6.53% (23 of 352 patients) lower incidence in SGLT2i users than in DPP4i users (*P* = .045).

**Conclusions and Relevance:**

The study findings suggest that patients with T2D who receive SGLT2i may have lower risk of AKI and AKI-D compared with those who receive DPP4i.

## Key Points

**Question:**

Is the use of sodium-glucose transport protein 2 inhibitors (SGLT2is) associated with acute kidney injury (AKI) in patients with type 2 diabetes?

**Findings:**

This cohort study of 104 462 patients with diabetes in Taiwan found that SGLT2i use vs dipeptidyl peptidase 4 inhibitor (DPP4i) use was associated with reductions of 34% and 44% in the risk of AKI and AKI requiring dialysis. Patients receiving SGLT2is had a lower incidence of AKI with respiratory failure and shock and a lower 90-day risk of advanced chronic kidney disease compared with those using DPP4is.

**Meaning:**

Our findings suggest that patients with type 2 diabetes who receive SGLT2i may have lower risk of AKI and AKI requiring dialysis than those who receive DPP4i.

## Teaser

This cohort study investigates the association of sodium-glucose transport protein 2 inhibitor (SGLT2i) use and the incidence of acute kidney injury (AKI) in patients with type 2 diabetes in Taiwan.

## Introduction

Acute kidney injury (AKI) is a syndrome with various etiologies that leads to decreased kidney function. AKI incidence has been increasing over time,^1^ resulting in short-term adverse outcomes, economic impact, and increased risk of chronic kidney disease (CKD), end-stage kidney disease (ESKD), and death.^2^ The prognosis of AKI that requires dialysis (AKI-D) is far worse than AKI, with short-term mortality rates often exceeding 50%^3^ and high long-term ESKD and mortality rates, even in patients undergoing temporary dialysis.^4^

Type 2 diabetes (T2D) was often associated with an increased risk of AKI^5^ and AKI-related morbidity and mortality,^6^ even without developing CKD. T2D was also a predictor of recurrent AKI and a risk factor for progressing to CKD.^7^ Although various strategies have been evaluated to mitigate the risk of AKI, no effective pharmacological agents can prevent AKI in patients with T2D.

Sodium-glucose cotransporter 2 inhibitors (SGLT2is) can control blood glucose and prevent cardiovascular and diabetic kidney disease progression in patients with T2D. Despite the warning of the US Food and Drug Administration Adverse Event Reporting System regarding the association between SGLT2i and the risk of AKI, an increasing number of clinical trials^8,9^ and clinical database studies^10,11^ have found that SGLT2i use was associated with a decreased risk of AKI. However, studies exploring the association between SGLT2i and AKI-D as well as concomitant diseases with AKI are lacking.

This cohort study used a Taiwan National Health Insurance data set to compare SGLT2i and dipeptidyl peptidase 4 inhibitors (DPP4i) use and the incidence of AKI and AKI-D in patients with T2D. Furthermore, we explored the associations between the 2 treatments and concomitant diseases with AKI along with the 90-day prognosis.

## Methods

### Data Sources

Data from the Taiwan National Health Insurance Research Database (NHIRD) was used. In 1995, a database comprising medical information of approximately 23 million individuals in Taiwan was constructed using the National Health Insurance program, recording claims for medical expenses, including outpatient and in-hospital care, laboratory tests, drug prescriptions, and interventional procedures. Taiwan’s National Health Research Institute permits access to the database for research purposes. Patient consent for accessing the NHIRD is not required. The China Medical University Hospital Ethics Committee approved this study, which was performed following the Declaration of Helsinki.^12^ Our study followed the Strengthening the Reporting of Observational Studies in Epidemiology (STROBE) reporting guidelines.

### Study Design and Participants

**Figure 1.**
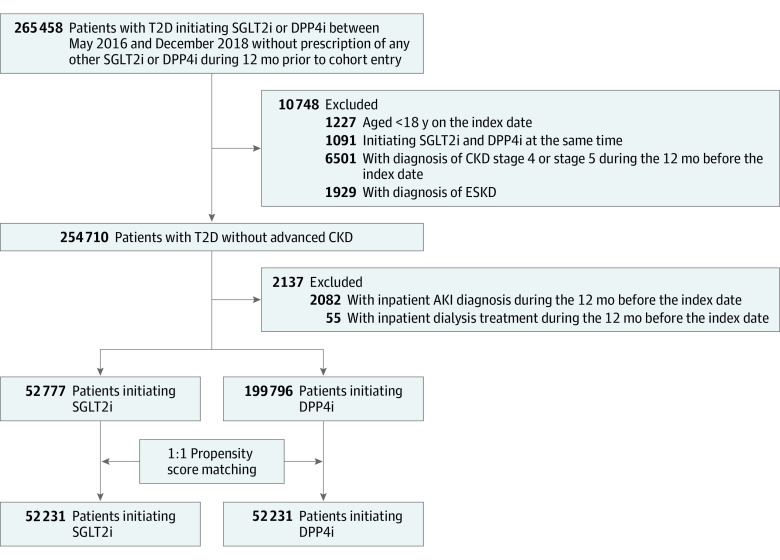
Diagram Showing the Study Flow and Patient Selection AKI indicates acute kidney disease; CKD, chronic kidney disease; DPP4i, dipeptidyl peptidase 4 inhibitor; ESKD, end-stage kidney disease; SGLT2i, sodium-glucose transporter protein 2 inhibitor; T2D, type 2 diabetes.

This was a nationwide retrospective cohort study with deidentified secondary data from the NHIRD. We used diagnostic codes from the *International Classification of Diseases, Ninth Revision, Clinical Modification *(*ICD-9-CM*) and *International Statistical Classification of Diseases, Tenth Revision, Clinical Modification (ICD-10-CM) *(eTable 1 in Supplement 1) to detect all patients with T2D. At least 3 outpatient clinic visits or 1 visit with hospitalization within 1 year were needed for T2D diagnosis, the accuracy of which was verified using the database.^13^ We initially located 265 458 patients with T2D who received SGLT2is or DPP4is between May 2016 (the SGLT2i release date in Taiwan) and December 2018. The index study date was defined as the date of first SGLT2i or DPP4i use. After excluding individuals younger than 18 years, simultaneous SGLT2i and DPP4i use before entry into the study, and those diagnosed with CKD stage 4 or 5 or ESKD for whom SGLT2i was contraindicated, 254 710 patients using SGLT2is or DPP4is without advanced CKD were included for initiation therapy. We also excluded patients with a history of inpatient AKI or dialysis, leaving 52 777 patients using SGLT2is and 199 796 using DPP4is in the cohort. Furthermore, we performed a 1:1 propensity score matching on 52 231 pairs of patients with T2D receiving SGLT2i or DPP4i based on age, sex, index year, comorbidities, and use of other drugs to avoid the confounding effects of baseline comorbidities and medication use. A detailed study flowchart is presented in Figure 1.

### SGLT2i or DPP4i Administration and AKI Outcomes

SGLT2i (dapagliflozin, empagliflozin, and canagliflozin) and DPP4i (alogliptin, linagliptin, sitagliptin, saxagliptin, and vildagliptin) were analyzed for drug type, quantity, dose, dispensing date, and days of drug supply. The primary outcomes were the incidences of AKI and AKI-D in the propensity score–matched cohort. AKI diagnosis was based on *ICD-9-CM* and *ICD-10-CM* diagnostic codes (eTable 1 in Supplement 1), while AKI-D diagnosis also required dialysis treatment during the same hospitalization. The dialysis treatment procedure codes are shown in eTable 1 in Supplement 1. The codes used to identify AKI were validated in our database, with a positive predictive value of 98.5% and a negative predictive value of 74.0%.^14^ The accuracy of acute dialysis procedure coding has also been validated, with a positive predictive value of 98%,^15^ as accurate procedure codes are necessary for reimbursement in Taiwan.

Different diseases interact with AKI and possibly aggravate it. Likewise, AKI can induce injury in these distant organs. These are grouped as AKI with heart disease, sepsis, respiratory failure, and shock. These 4 diseases were chosen because they are the most common contributors to AKI.^16^ These diseases were diagnosed based on *ICD-9-CM* and *ICD-10-CM* diagnostic codes (eTable 1 in Supplement 1) during the AKI hospitalization. AKI prognosis was also analyzed. We considered advanced CKD (defined as CKD stages 4 and 5 by *ICD-10-CM* diagnostic codes), ESKD (confirmed by the registry of catastrophic illness), or death that occurred within 90 days of AKI hospitalization.

### Covariates

The distributions of baseline comorbidities and prescription histories in the SGLT2i and DPP4i groups were compared. Comorbidities included hypertension, hyperlipidemia, cerebral vascular disease (CVD), coronary artery disease (CAD), and CKD that occurred within 1 year before the index date. In addition to the use of SGLT2is or DPP4is, our models considered other medications used during the year before the index date, such as glucagonlike peptide-1 (GLP-1) agonists, insulin, metformin, diuretics, statins, aspirin, angiotensin-converting enzyme inhibitors (ACEIs)/angiotensin receptor blocker (ARBs), nonsteroidal anti-inflammatory drugs (NSAIDs), and proton pump inhibitors (PPIs).

### Statistical Analysis

We performed propensity score matching to construct a comparative cohort for SGLT2i users from the study population. Propensity scores (PS) were calculated as a probability dependent on a vector of observed covariates associated with receipt of treatment with SGLT2i. We conducted a logistic regression analysis for estimating PS with adjustment for age, sex, comorbidities, and related clinical medications. A 1:1 PS-matched cohort was constructed using greedy nearest neighbor matching, and the caliper width was within 0.2.^17^ Moreover, standardized mean differences with a cutoff value of 0.10 were used to observe the fitness of covariate comparisons between the propensity score–matched groups.^18,19^ Continuous variables are presented as mean and SD and categorical variables as numbers and frequencies. Age was stratified into 10-year groups of younger than 25 years, 25 to 34 years, 35 to 44 years, and so on. All participants were followed up from the index date until AKI diagnosis, death, or the study end date (December 31, 2018), whichever occurred first. Kaplan-Meier survival curves were expressed and compared for the risk of AKI or AKI-D incidence using log-rank tests. We used conditional Cox proportional hazard regressions to determine the crude and adjusted hazard ratios (HRs) and 95% CIs for risk of AKI or AKI-D after SGLT2i administration. SGLT2is, including dapagliflozin, empagliflozin, and canagliflozin, were evaluated separately for AKI or AKI-D risk using stratification analysis. We then added a multiplicative interaction term to the regression models to calculate the interactions between comorbidities and the use of SGLT2i on AKI risk. Finally, we analyzed the associations between SGLT2i use and concomitant diseases with AKI using conditional Cox proportional hazard regression analysis. The prognostic outcomes of AKI, including advanced CKD, ESKD, and death, were compared using χ^2^ tests. All hypothesis tests were 2-sided. Significance was defined as α = 0.05. Statistical analysis was performed using the SAS statistical software version 9.4 (SAS Institute). Data analysis was conducted from October 15, 2021, to January 30, 2022.

## Results

### Characteristics of the Study Participants

Of the 252 573 individuals with T2D, 52 777 (20.9%) received SGLT2is (Table 1). Compared with DPP4i users, SGLT2i users were younger, more likely to be male, had hyperlipidemia or CAD, and received diabetes-related medications, diuretics, statins, aspirin, ACEIs/ARBs, NSAIDs, and PPIs. We used propensity score matching to select 52 231 pairs receiving SGLT2i or DPP4i to avoid the confounding effects of the previously mentioned variables.

**Table 1.** Demographic Profiles of Patients With Type 2 Diabetes Using DPP4i and SGLT2i in the Overall Study Population and the Propensity Score–Matched Population

Characteristic	Full study population			SMD	Propensity score–matched cohort			SMD
	Overall (N = 252 573)	DPP4i (N = 199 796)	SGLT2i (N = 52 777)		Overall (N = 104 462)	DPP4i (N = 52 231)	SGLT2i (N = 52 231)	
Age, y								
Mean (SD)	61.48 (12.84)	62.49 (12.81)	57.66 (12.24)	−0.39	57.75 (12.32)	57.72 (12.45)	57.78 (12.19)	0.01
<25	882 (0.35)	579 (0.29)	303 (0.57)		658 (0.63)	366 (0.70)	292 (0.56)	
25-34	4582 (1.81)	3030 (1.52)	1552 (2.94)		3047 (2.92)	1585 (3.03)	1462 (2.80)	
35-44	19 750 (7.82)	13 598 (6.81)	6152 (11.66)		11 977 (11.47)	5990 (11.47)	5987 (11.46)	
45-54	45 618 (18.06)	33 906 (16.97)	11 712 (22.19)		23 144 (22.16)	11 564 (22.14)	11 580 (22.17)	
55-64	77 630 (30.74)	60 247 (30.15)	17 383 (32.94)		34 354 (32.89)	17 075 (32.69)	17 279 (33.08)	
65-74	63 126 (24.99)	51 639 (25.85)	11 487 (21.77)		22 601 (21.64)	11 156 (21.36)	11 445 (21.91)	
75-84	32 530 (12.88)	28 865 (14.45)	3665 (6.94)		7604 (7.28)	3941 (7.55)	3663 (7.01)	
≥85	8455 (3.35)	7932 (3.97)	523 (0.99)		1077 (1.03)	554 (1.06)	523 (1.00)	
Sex								
Women	118 998 (47.11)	95 829 (47.96)	23 169 (43.90)	0.08	46 065 (44.10)	23 120 (44.26)	22 945 (43.93)	0.01
Men	133 575 (52.89)	103 967 (52.04)	29 608 (56.10)		58 397 (55.90)	29 111 (55.74)	29 286 (56.07)	
Comorbidities								
Hypertension	26 914 (10.66)	22 108 (11.07)	4806 (9.11)	0.07	8830 (8.45)	4085 (7.82)	4745 (9.08)	−0.05
Hyperlipidemia	170 082 (67.34)	132 371 (66.25)	37 711 (71.45)	−0.11	74 891 (71.69)	37 597 (71.98)	37 294 (71.40)	0.01
Cerebral vascular disease	4695 (1.86)	4162 (2.08)	533 (1.01)	0.09	933 (0.89)	401 (0.77)	532 (1.02)	−0.03
Coronary artery disease	43 189 (17.10)	32 677 (16.36)	10 512 (19.92)	−0.09	20 095 (19.24)	9741 (18.65)	10 354 (19.82)	−0.03
Chronic kidney disease	26 488 (10.49)	21 280 (10.65)	5208 (9.87)	0.03	9638 (9.23)	4506 (8.63)	5132 (9.83)	−0.04
Diabetes medications								
GLP-1 agonist	1240 (0.49)	371 (0.19)	869 (1.65)	−0.15	886 (0.85)	354 (0.68)	532 (1.02)	−0.04
Insulin	30 708 (12.16)	20 274 (10.15)	10 434 (19.77)	−0.27	19 516 (18.68)	9502 (18.19)	10 014 (19.17)	−0.03
Metformin	187 715 (74.32)	140 282 (70.21)	47 433 (89.87)	−0.51	94 374 (90.34)	47 482 (90.91)	46 892 (89.78)	0.04
Other medications								
ACEI/ARB	127 391 (50.44)	98 754 (49.43)	28 637 (54.26)	−0.10	56 348 (53.94)	28 068 (53.74)	28 280 (54.14)	−0.01
Diuretics	22 253 (8.81)	17 328 (8.67)	4925 (9.33)	−0.02	9143 (8.75)	4296 (8.23)	4847 (9.28)	−0.04
Statin	138 431 (54.81)	105 843 (52.98)	32 588 (61.75)	−0.18	64 652 (61.89)	32 477 (62.18)	32 175 (61.60)	0.01
Aspirin	55 876 (22.12)	42 777 (21.41)	13 099 (24.82)	−0.08	25 186 (24.11)	12 272 (23.50)	12 914 (24.72)	−0.03
PPI	10 657 (4.22)	8444 (4.23)	2213 (4.19)	0.00	4112 (3.94)	1933 (3.70)	2179 (4.17)	−0.02
NSAIDs	86 768 (34.35)	67 493 (33.78)	19 275 (36.52)	−0.06	38 238 (36.60)	19 189 (36.74)	19 049 (36.47)	0.01

Abbreviations: ACEI, angiotensin-converting enzyme inhibitor; ARB, angiotensin receptor blocker; DPP4i, dipeptidyl peptidase 4 inhibitor; GLP-1, glucagonlike peptide-1; NSAIDs, nonsteroidal anti-inflammatory drugs; PPI, proton pump inhibitors; SGLT2i, sodium-glucose transport protein 2 inhibitor; SMD, standardized mean difference.

### SGLT2i Use and the Risk of AKI

**Figure 2.**
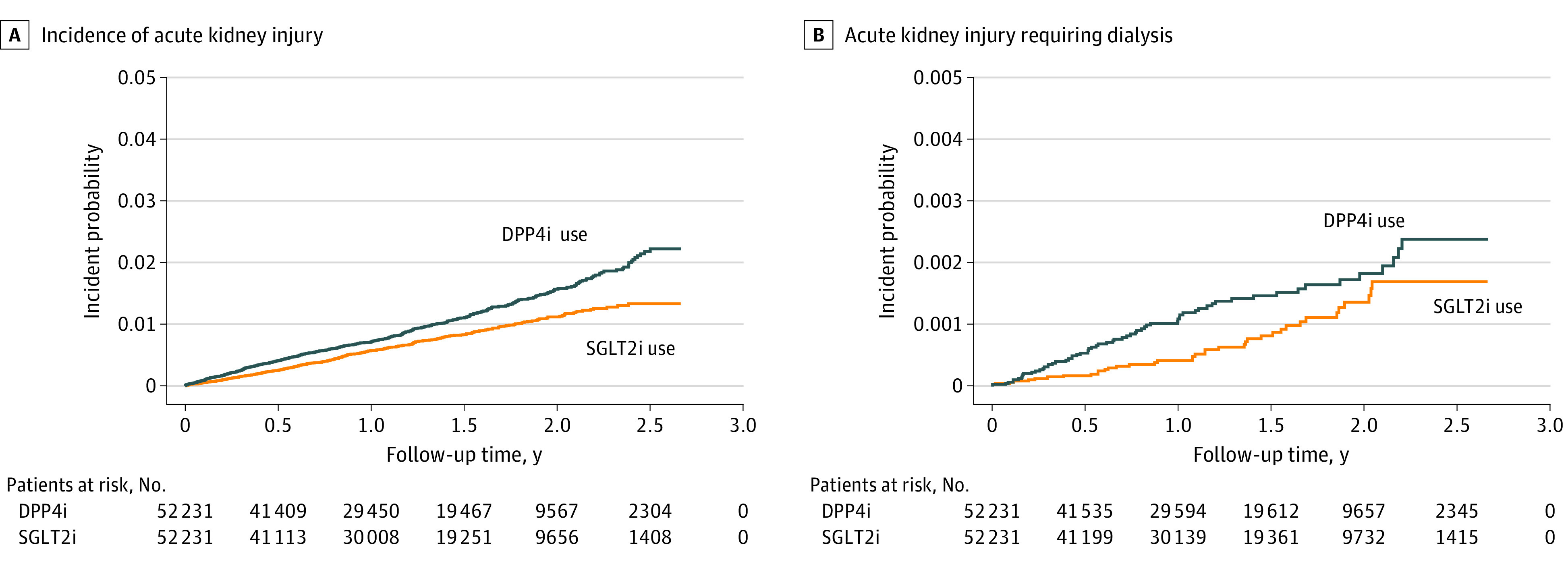
Kaplan-Meier Curves Showing the Incidences of Acute Kidney Disease and Acute Kidney Disease Requiring Dialysis in Patients With Type 2 Diabetes Using Dipeptidyl Peptidase 4 Inhibitors (DPP4is) or Sodium-Glucose Transporter Protein 2 Inhibitors (SGLT2i)

Of the propensity score–matched cohort (n = 104 462), 46 065 (44.1%) were female patients, and the mean (SD) age was 58 (12) years. Overall, 856 participants (0.8%) had AKI and 102 (<0.1%) had AKI-D during the study period. Log-rank tests indicated that SGLT2i users had lower cumulative incidences of AKI (SGLT2i vs DPP4i users: 5.55 per 1000 patient-years vs 7.88 per 1000 patient-years; *P* < .001) (Figure 2A) and AKI-D (SGLT2i vs DPP4i users: 5.98 per 10 000 patient-years vs 9.96 per 10 000 patient-years; *P* = .01) (Figure 2B). After adjusting for age, sex, hypertension, hyperlipidemia, CVD, CAD, CKD, and use of GLP-1 agonists, insulin, metformin, diuretics, statins, aspirin, ACEIs/ARBs, NSAIDs, and PPIs, the SGLT2i group had an adjusted HR of 0.66 (95% CI, 0.57-0.75; *P* < .001) for AKI and 0.56 (95% CI, 0.37-0.84; *P* = .005) for AKI-D compared with the DPP4i group (Table 2). The most commonly used SGLT2i was dapagliflozin (29 116 [55.7%]), followed by empagliflozin (22 526 [43.1%]) and canagliflozin (589 [1.1%]). The association of SGLT2i use with reduced risk of AKI and AKI-D was found in dapagliflozin and empagliflozin users. Analysis of the associations between SGLT2i use and AKI and AKI-D risk in all study populations is additionally shown in eTable 2 in Supplement 1. Furthermore, demographic profiles of dapagliflozin and empagliflozin users in the propensity score–matched population are provided in eTable 3 in Supplement 1. Compared with empagliflozin users, dapagliflozin users were younger; more likely to be women; had a lower prevalence of hypertension, CVD, CAD, and CKD; and were prescribed fewer medications except for metformin and NSAIDs.

**Table 2.** Incidence and Risk of AKI and AKI-D Between Patients Receiving DPP4is and SGLT2is in the Propensity Score–Matched Cohort of Patients With Type 2 Diabetes

Outcome	Events, No.	Person years	Incident rate^a^	Crude HR (95% CI)	*P* value	Adjusted HR (95% CI)^b^	*P* value
AKI							
DPP4i (n = 52 231)	504	63 944.56	7.88	1 [Reference]	NA	1 [Reference]	NA
SGLT2i (n = 52 231)	352	63 374.28	5.55	0.71 (0.62-0.81)	<.001	0.66 (0.57-0.75)	<.001
Canagliflozin (n = 589)	2	174.51	11.46	1.46 (0.36-5.87)	.59	1.41 (0.35-5.58)	.63
Dapagliflozin (n = 29 116)	171	35 725.04	4.79	0.61 (0.51-0.72)	<.001	0.61 (0.52-0.73)	<.001
Empagliflozin (n = 22 526)	179	27 474.74	6.52	0.83 (0.70-0.98)	.03	0.70 (0.59-0.84)	<.001
AKI-D							
DPP4i (n = 52 231)	64	64 225.28	1.00	1 [Reference]	NA	1 [Reference]	NA
SGLT2i (n = 52 231)	38	63 582.81	0.60	0.60 (0.40-0.89)	.012	0.56 (0.37-0.84)	.005
Canagliflozin (n = 589)	0	174.67	0.00	NA	NA	NA	NA
Dapagliflozin (n = 29 116)	19	35 823.25	0.53	0.53 (0.32-0.89)	.02	0.54 (0.32-0.90)	.02
Empagliflozin (n = 22 526)	19	27 584.88	0.69	0.69 (0.41-1.15)	.15	0.59 (0.35-0.99)	.04

Abbreviations: AKI, acute kidney injury; AKI-D, AKI with dialysis; DPP4i, dipeptidyl peptidase 4 inhibitors; HR, hazard ratio; NA, not applicable; SGLT2i, sodium-glucose transport protein 2 inhibitors.

^a^ Incident rates were calculated as events per 1000 person-years.

^b^ Adjusted HRs were calculated by adjusting for age, sex, hypertension, hyperlipidemia, cerebral vascular disease, coronary artery disease, chronic kidney disease, and use of glucagonlike peptide-1 agonists, insulin, metformin, angiotensin-converting enzyme inhibitors/angiotensin receptor blockers, diuretics, statins, aspirin, proton pump inhibitors, and nonsteroidal anti-inflammatory drugs.

Because of the low AKI-D incidence, we only assessed the interaction between the comorbidities and SGLT2i use and AKI risk in the propensity score–matched population (eTable 4 in Supplement 1). A protective association for SGLT2i use against AKI was observed in patients with T2D but without hypertension or CVD. We noted no obvious interactions between these comorbidities and SGLT2i use on the risk of AKI. Concurrent use of SGLT2i and metformin resulted in an insignificantly greater reduction in AKI risk than SGLT2i alone. Significant protective associations between SGLT2i and AKI risk were noted regardless of insulin, diuretic, statin, aspirin, ACEI/ARB, NSAID, or PPI use.

**Figure 3.**
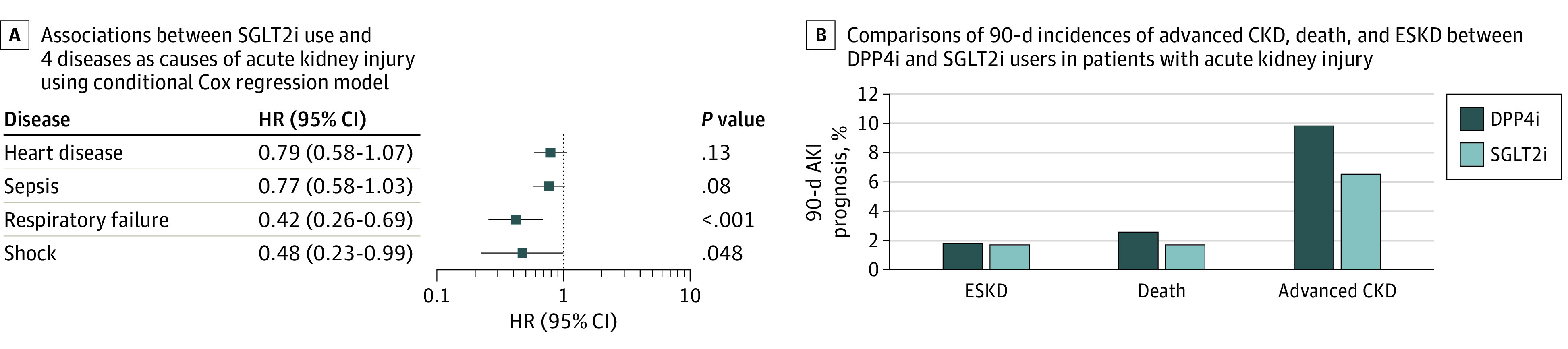
Associations Between Sodium-Glucose Transporter Protein 2 Inhibitor (SGLT2i) Use and 4 Diseases as Causes of Acute Kidney Injury and Comparisons of 90-Day Incidence of Advanced Chronic Kidney Disease (CKD), Death, and End-Stage Kidney Disease (ESKD) DPP4i indicates dipeptidyl peptidase 4 inhibitor; HR, hazard ratio.

Among patients with AKI, heart disease, sepsis, respiratory failure, and shock occurred in 80 (22.73%), 83 (23.58%), 23 (6.53%), and 10 (2.84%), respectively. Figure 3A shows significant benefits were associated with SGLT2i use in AKI with respiratory failure (HR, 0.42; 95% CI, 0.26-0.69; *P* < .001) and shock (HR, 0.48; 95% CI, 0.23-0.99; *P* = .048) but not AKI with heart disease (HR, 0.79; 95% CI, 0.58-1.07; *P* = .13) and sepsis (HR, 0.77; 95% CI, 0.58-1.03; *P* = .08).

Of the 856 patients with AKI, 66 (7.7%) had advanced CKD, 15 (1.8%) had ESKD, and 19 (2.2%) died within 90 days. The 90-day prognosis for the risk of advanced CKD indicated a 6.53% (23 of 352 patients) lowered incidence among SGLT2i users compared with DPP4i users (*P* = .045). The incidences of ESKD and death among SGLT2i and DPP4i users were similar.

## Discussion

This nationwide population-based study of propensity score–matched adults showed that SGLT2i use was associated with a lower risk of AKI (34%) and AKI-D (44%) than DPP4i in patients with T2D. To our knowledge, this is the first study to show that SGLT2i users had a lower risk of concomitant diseases with AKI, including AKI with shock and respiratory failure. Furthermore, patients treated with SGLT2is had a lower 90-day incidence of advanced CKD following AKI.

Our study showed that SGLT2i was associated with a lower incidence of AKI. It was important to investigate which glucose-lowering drugs could protect patients with T2D from AKI because T2D was associated with a higher risk of AKI.^5^ Early and recent meta-analyses of clinical trials^8,9^ suggested that SGLT2i use was associated with a 16% to 25% reduced risk of AKI compared with placebo. Furthermore, a network meta-analysis^20^ showed that the risk of AKI in SGLT2i users was 21% and 32% lower than in GLP-1 agonist and DPP4i users, respectively. However, AKI is considered an adverse event rather than a prespecified outcome in clinical trials. Our study provides more evidence of this association between SGLT2i use and the lower incidence of AKI.

AKI-D is another crucial outcome because it was associated with in-hospital and long-term mortality^4^ and numerous comorbidities.^14,21^ To our knowledge, only 1 large registry study had indicated that patients with T2D using SGLT2i had a lower risk of AKI-D than those using DPP4i.^10^ Large studies are needed to achieve sufficient statistical power to detect a difference because AKI-D events are usually rare. Our findings indicated that SGLT2i users had fewer AKI and AKI-D events, which means less severity of AKI. Similarly, a previous study found that SGLT2i prescriptions were associated with less severe AKI based on the change in serum creatinine from baseline to the peak level.^11^

Several factors likely accounted for the association between SGLT2i administration and the lower risk and severity of AKI. First, decreased intraglomerular pressure when using SGLT2is is associated with lower podocyte stress and proteinuria, exerting the main kidney-protective effects.^22^ Second, magnetic resonance imaging has shown that SGLT2i improved renal cortical oxygenation^23^ through reduced oxygen consumption by decreasing sodium and glucose reabsorption in the proximal tubules, erythropoietin production, and hemoconcentration.^22^ SGLT2is could also protect kidney cells from hypoxic injury through cellular signaling and decrease inflammation.^24^ Finally, mounting basic research evidence suggests that SGLT2i use benefits sepsis-,^25^ heart failure-,^26^ contrast media-,^27^ and nephrotoxin-associated AKI.^28^

AKI is a syndrome that rarely has a sole distinct pathophysiology cause. Our study used the discharge codiagnosis of AKI and sepsis, heart disease, shock, or respiratory failure. SGLT2i use was associated with a significantly lower incidence of AKI with shock and respiratory failure. An association between SGLT2i use and a lower risk of AKI with sepsis and heart disease was observed. A meta-analysis of heart failure trials showed that SGLT2i use was associated with a lower risk of a composite kidney end point.^29^ Patients with acute decompensated heart failure taking empagliflozin had lower AKI markers than those who did not.^30^ A recent meta-analysis of acute heart failure trials also detected an association between reduced AKI risk and SGLT2i use.^31^ Basic research in a rat model has also shown that SGLT2i attenuates AKI after myocardial infarction by alleviating oxidative stress.^26^ To our knowledge, the associations between SGLT2i and a lower risk of AKI with shock and respiratory failure have not yet been reported. Further studies are needed to confirm these findings.

A meta-analysis showed that the incidence of de novo CKD or CKD progression in patients with AKI was higher than in those without.^32^ Acute kidney disease was proposed to reflect the continuum between AKI and CKD, with subacute damage of 7 to 90 days after AKI.^33^ We assessed 90-day events, including advanced CKD, ESKD, and death, showing that SGLT2i use was associated with a lower risk of advanced CKD. There are several possible explanations for this finding. As mentioned previously, patients using SGLT2i had less severe AKI, and a previous study showed that AKI severity determined the risk of CKD progression.^32^ Furthermore, SGLT2i was shown to attenuate kidney capillary injury and fibrosis in an ischemic reperfusion injury mouse model through increased vascular endothelial growth factor expression.^34^

Our study’s strengths include its large sample and nationwide scope, with more than 99% coverage of approximately 23 million individuals in Taiwan. A large sample was needed to explore rare outcomes such as AKI-D. The diagnostic accuracy of T2D and AKI in the health insurance claims data in Taiwan has been validated. To our knowledge, our study is the first to propose the probable protective benefit of SGLT2i use against concomitant diseases with AKI and AKI prognosis in a real-world database.

### Limitations

Our study has limitations. First, we could not identify the baseline glomerular filtration rate and detailed laboratory values from the *ICD* codes, making it difficult to identify the cause and the severity of CKD and AKI. Second, we used the *ICD* codes to identify AKI, possibly skewing the cohort toward severe AKI. Furthermore, we could not clarify the severity of acute disease and possible nephrotoxic drug use (eg, vasopressors) during hospitalization, which may affect AKI prognoses. Additionally, although we used propensity score matching to balance potential confounders between SGLT2i and DPP4i users, confounding by indication could still be present.

## Conclusions

In this study, SGLT2i use among patients with T2D was associated with a lower risk of AKI, decreased AKI severity, and improved prognosis compared with DPP4i. These findings suggest that SGLT2is may be an effective way to prevent AKI and AKI-D and improve outcomes.

## Notes

Supplement 1. **eTable 1.** Diagnostic Codes of Various Diseases From *ICD-9-CM* and *ICD-10-CM* **eTable 2.** Incidence and Risk of AKI Among Patients Using DPP4is and SGLT2is With Type 2 Diabetes in Full Study Population **eTable 3.** Demographic Profiles of Patients With Type 2 Diabetes Using Dapagliflozin and Empagliflozin in the Propensity Score–Matched Population **eTable 4.** Stratified and Interaction Analysis of AKI Incidence in Patients With Type 2 Diabetes Receiving Dipeptidyl Peptidase 4 (DPP4) Inhibitors and Sodium-Glucose Cotransporter 2 (SGLT2) Inhibitors Using Baseline Comorbidities and Other Medications

Supplement 2.
Data Sharing Statement
