# Supplementary material for: Sodium-Glucose Transport Protein 2 Inhibitor Use for Type 2 Diabetes and the Incidence of Acute Kidney Injury in Taiwan
Source: JAMA Netw Open. 2023 Feb 22;6(2):e230453. doi: 10.1001/jamanetworkopen.2023.0453 (PMC9947724; doi:10.1001/jamanetworkopen.2023.0453)
Supplement: Supplement 1. — eTable 1. Diagnostic Codes of Various Diseases From ICD-9-CM and ICD-10-CM eTable 2. Incidence and Risk of AKI Among Patients Using DPP4is and SGLT2is With Type 2 Diabetes in Full Study Population eTable 3. Demographic Profiles of Patients With Type 2 Diabetes Using Dapagliflozin and Empagliflozin in the Propensity Score–Matched Population eTable 4. Stratified and Interaction Analysis of AKI Incidence in Patients With Type 2 Diabetes Receiving Dipeptidyl Peptidase 4 (DPP4) Inhibitors and Sodium-Glucose Cotransporter 2 (SGLT2) Inhibitors Using Baseline Comorbidities and Other Medications [file jamanetwopen-e230453-s001.pdf]

Supplemental Online Content

Chung MC, Hung PH, Hsiao PJ, et al. Sodium-glucose transport protein 2 inhibitor use for type 2 diabetes and the incidence of acute kidney injury in Taiwan. *JAMA Netw Open.* 2023;6(2):e230453. doi:10.1001/jamanetworkopen.2023.0453

eTable 1. Diagnostic Codes of Various Diseases From *ICD-9-CM* and *ICD-10-CM*

eTable 2. Incidence and Risk of AKI Among Patients Using DPP4is and SGLT2is With Type 2 Diabetes in Full Study Population

eTable 3. Demographic Profiles of Patients With Type 2 Diabetes Using Dapagliflozin and Empagliflozin in the Propensity Score–Matched Population

eTable 4. Stratified and Interaction Analysis of AKI Incidence in Patients With Type 2 Diabetes Receiving Dipeptidyl Peptidase 4 (DPP4) Inhibitors and Sodium-Glucose Cotransporter 2 (SGLT2) Inhibitors Using Baseline Comorbidities and Other Medications

This supplemental material has been provided by the authors to give readers additional information about their work.

© 2023 Chung MC et al. *JAMA Network Open.*

eTable 1. Diagnostic Codes of Various Diseases From *ICD-9-CM* and *ICD-10-CM*

Type 2 Diabetes Mellitus	ICD-09: "250.0","250.00","250.02","250.10","250.12","250.2","250.20","250.22","250.30","250.32","250.40","250.42","250.50","250.52","250.60","250.62","250.70","250.72","250.80","250.82","250.90","250.92".<br><br>ICD-10: "E11","E110","E110.0","E110.1","E112","E112.1","E112.2","E112.9","E113","E113.1","E113.11","E113.19","E113.2","E113.21","E113.29","E113.3","E113.31","E113.39","E113.4","E113.41","E113.49","E113.5","E113.51","E113.59","E113.6","E113.9","E114","E114.0","E114.1","E114.2","E114.3","E114.4","E114.9","E115","E115.1","E115.2","E115.9","E116","E116.1","E116.10","E116.18","E116.2","E116.20","E116.21","E116.22","E116.28","E116.3","E116.30","E116.38","E116.4","E116.41","E116.49","E116.5","E118","E119".
Acute kidney injury	ICD-09: “584.X”<br>ICD-10: “N17.X”
Dialysis	58001C, 58019C, 58020C, 58021C, 58022C, 58023C, 58024C, 58025C, 58029C, 58002C, 58009A, 58009B, 58010A, 58010B, 58011A, 58011AB, 58011B, 58011C, 58012A, 58012B, 58017B, 58017C, 58026C, 58028C, 58018C, 58027C, 58030B, 58007C, 58014C
Heart disease	ICD-10: I00-I09, I11, I13, I20-I51
Sepsis	ICD-10: A40-A41
Respiratory failure	ICD-10: J96
Shock	ICD-10: R57
Advanced chronic kidney disease	ICD-10: N18.4 for stage 4.<br>ICD-10: N18.5 for stage 5.

© 2023 Chung MC et al. *JAMA Network Open.*

eTable 2. Incidence and Risk of AKI Among Patients Using DPP4is and SGLT2is With Type 2 Diabetes in Full Study Population							
	Events, n	Person years	Incident rate	Crude HR (95%CI)	p-value	Adjusted HR (95%CI)	p-value
AKI							
DPP4i (N = 199,796)	2,921	267416.83	10.92	REF		REF	
SGLT2i (N = 52,777)	352	63963.23	5.50	0.50 (0.45 - 0.56)	<.001	0.66 (0.58 - 0.74)	<.001
Canagliflozin (N = 595)	2	176.57	11.33	1.06 (0.27 - 4.24)	.93	1.43 (0.36 - 5.74)	.61
Dapagliflozin (N = 29,419)	171	36072.51	4.74	0.43 (0.37 - 0.51)	<.001	0.61 (0.52 - 0.71)	<.001
Empagliflozin (N = 22,763)	179	27714.16	6.46	0.59 (0.51 - 0.69)	<.001	0.70 (0.60 - 0.82)	<.001
AKI-D							
DPP4i (N = 199,796)	338	269160.79	1.26	REF		REF	
SGLT2i (N = 52,777)	38	64171.76	0.59	0.48 (0.34 - 0.66)	<.001	0.57 (0.40 - 0.80)	.001
Canagliflozin (N = 595)	0	176.74	0.00	-	-	-	-
Dapagliflozin (N = 29,419)	19	36170.72	0.53	0.42 (0.27 - 0.67)	<.001	0.55 (0.34 - 0.87)	.01
Empagliflozin (N = 22,763)	19	27824.30	0.68	0.55 (0.35 - 0.87)	.01	0.60 (0.37 - 0.96)	.03
Incident rates were calculated as events of gout per 1000 person-years. AKI-D: AKI requiring dialysis							
Adjusted HR were calculated by adjusting for age, gender, HTN, HPL, CVD, CAD, CKD, GLP-1 agonist, insulin, metformin, ACEI / ARB, diuretics, statin, aspirin, PPI and NSAIDs							

© 2023 Chung MC et al. *JAMA Network Open.*

eTable 3. Demographic Profiles of Patients With Type 2 Diabetes Using Dapagliflozin and Empagliflozin in the Propensity Score–Matched Population		
	Dapagliflozin	Empagliflozin
	(N=29,116)	(N=22,526)
Age, Mean ± SD	57.17 ± 12.09	58.55 ± 12.27
Stratify age, n (%)		
<25	163 (0.56%)	124 (0.55%)
25-34	882 (3.03%)	567 (2.52%)
35-44	3,537 (12.15%)	2,391 (10.61%)
45-54	6,718 (23.07%)	4,729 (20.99%)
55-64	9,698 (33.31%)	7,386 (32.79%)
65-74	6,059 (20.81%)	5,258 (23.34%)
75-84	1,831 (6.29%)	1,784 (7.92%)
>=85	228 (0.78%)	287 (1.27%)
Gender (Women), n (%)	13,112 (45.03%)	9,575 (42.51%)
Comorbidity, n (%)		
Hypertension	2,276 (7.82%)	2,407 (10.69%)
Hyperlipidemia	20,964 (72.00%)	15,912 (70.64%)
Cerebral vascular disease	238 (0.82%)	287 (1.27%)
Coronary artery disease	5,248 (18.02%)	4,988 (22.14%)
Chronic kidney disease	2,577 (8.85%)	2,484 (11.03%)
Diabetes medications, n (%)		
GLP-1 agonist	285 (0.98%)	238 (1.06%)
Insulin	5,200 (17.86%)	4,724 (20.97%)
Metformin	26,349 (90.50%)	20,015 (88.85%)
Other medications, n (%)		
ACEI / ARB	15,228 (52.30%)	12,714 (56.44%)
Diuretics	2,437 (8.37%)	2,356 (10.46%)
Statin	17,787 (61.09%)	13,994 (62.12%)
Aspirin	6,654 (22.85%)	6,125 (27.19%)
PPI	1,110 (3.81%)	1,046 (4.64%)
NSAIDs	10,728 (36.85%)	8,084 (35.89%)

GLP-1: glucagonlike peptide-1; ACEI: angiotensin-converting enzyme inhibitor; ARB: angiotensin receptor blocker; NSAIDs: non-steroidal anti-inflammatory drugs; PPI: proton pump inhibitors

© 2023 Chung MC et al. *JAMA Network Open.*

eTable 4. Stratified and Interaction Analysis of AKI Incidence in Patients With Type 2 Diabetes Receiving Dipeptidyl Peptidase 4 (DPP4) Inhibitors and Sodium-Glucose Cotransporter 2 (SGLT2) Inhibitors Using Baseline Comorbidities and Other Medications

	Propensity Score–Matched Population		
	Events, n	HR(95%CI)	interaction p
Acute kidney injury			
Age			0.10
<65	154	0.58 (0.47 - 0.70)	
>=65	198	0.74 (0.61 - 0.89)	
Gender			0.19
Male	198	0.62 (0.51 - 0.74)	
Female	154	0.73 (0.59 - 0.91)	
Comorbidity			
Hypertension			0.10
No	264	0.62 (0.53 - 0.72)	
Yes	88	0.82 (0.61 - 1.11)	
Hyperlipidemia			0.89
No	127	0.65 (0.52 - 0.82)	
Yes	225	0.66 (0.56 - 0.78)	
Cerebral vascular disease			0.89
No	345	0.66 (0.57 - 0.76)	
Yes	7	0.78 (0.30 - 2.06)	
Coronary artery disease			0.87
No	239	0.65 (0.55 - 0.77)	
Yes	113	0.68 (0.53 - 0.88)	
Chronic kidney disease			0.81
No	279	0.65 (0.56 - 0.76)	
Yes	73	0.68 (0.50 - 0.93)	
Diabetes medications			
GLP-1 agonist			0.85
No	346	0.66 (0.57 - 0.75)	
Yes	6	0.51 (0.10 - 2.60)	
Insulin			0.07
No	240	0.72 (0.61 - 0.85)	
Yes	112	0.55 (0.44 - 0.70)	
Metformin			0.11
No	72	0.88 (0.61 - 1.26)	
Yes	280	0.63 (0.54 - 0.73)	
Other medications			

© 2023 Chung MC et al. *JAMA Network Open.*

ACEI / ARB						0.98
No		123		0.65 (0.52 - 0.82)		
Yes		229		0.66 (0.56 - 0.79)		
Diuretics						0.33
No		268		0.68 (0.59 - 0.80)		
Yes		84		0.59 (0.44 - 0.78)		
Statin						0.96
No		159		0.65 (0.53 - 0.80)		
Yes		193		0.66 (0.55 - 0.80)		
Aspirin						0.51
No		228		0.63 (0.54 - 0.75)		
Yes		124		0.71 (0.56 - 0.90)		
PPI						0.17
No		333		0.68 (0.59 - 0.78)		
Yes		19		0.46 (0.26 - 0.81)		
NSAIDs						0.93
No		238		0.66 (0.56 - 0.78)		
Yes		114		0.65 (0.52 - 0.83)		

GLP-1: glucagonlike peptide-1; ACEI: angiotensin-converting enzyme inhibitor; ARB: angiotensin receptor blocker; NSAIDs: non-steroidal anti-inflammatory drugs; PPI: proton pump inhibitors

© 2023 Chung MC et al. *JAMA Network Open.*
