# Supplementary material for: Sodium-Glucose Transport Protein 2 Inhibitor Use for Type 2 Diabetes and the Incidence of Acute Kidney Injury in Taiwan
Source: JAMA Netw Open. 2023 Feb 22;6(2):e230453. doi: 10.1001/jamanetworkopen.2023.0453 (PMC9947724; doi:10.1001/jamanetworkopen.2023.0453)
Supplement: Supplement 2. — Data Sharing Statement [file jamanetwopen-e230453-s002.pdf]

Data Sharing Statement

Chung. Sodium-Glucose Transport Protein 2 Inhibitor Use for Type 2 Diabetes and the Incidence of Acute Kidney Injury in Taiwan. *JAMA Netw Open*. Published February 22, 2023. doi:10.1001/jamanetworkopen.2023.0453

Data
Data available: No
